# Impact of lymphadenectomy extent on immunotherapy efficacy in postresectional recurred non-small cell lung cancer: a multi-institutional retrospective cohort study

**DOI:** 10.1097/JS9.0000000000000774

**Published:** 2023-09-26

**Authors:** Hongsheng Deng, Juan Zhou, Hualin Chen, Xiuyu Cai, Ran Zhong, Feng Li, Bo Cheng, Caichen Li, Qingzhu Jia, Caicun Zhou, René H. Petersen, Gaetano Rocco, Alex Brunelli, Calvin S.H. Ng, Thomas A. D’Amico, Chunxia Su, Jianxing He, Wenhua Liang, Bo Zhu

**Affiliations:** a Department of Thoracic Surgery and Oncology, The First Affiliated Hospital of Guangzhou Medical University, State Key Laboratory of Respiratory Disease, National Clinical Research Center for Respiratory Disease, Guangzhou Institute of Respiratory Health, Guangzhou, China; b Department of Medical Oncology, Shanghai Pulmonary Hospital and Thoracic Cancer Institute, Tongji University School of Medicine, Shanghai, China; c Department of Pulmonary Oncology, Affiliated Hospital of Guangdong Medical University, Zhanjiang, China; d Department of Medical Oncology, Sun Yat-Sen University Cancer Center, State Key Laboratory of Oncology in South China, Collaborative Innovation Center for Cancer Medicine, Guangzhou, China; e Institute of Cancer, Xinqiao Hospital, The Army Medical University, Chongqing, China; f Department of Cardiothoracic Surgery, University Hospital of Copenhagen, Rigshospitalet, Copenhagen, Denmark; g Thoracic Service, Department of Surgery, Memorial Sloan Kettering Cancer Center, New York, USA; h Department of Thoracic Surgery, St. James's University Hospital, Leeds, UK; i Department of Surgery, Division of Cardiothoracic Surgery, Prince of Wales Hospital, The Chinese University of Hong Kong, Hong Kong SAR, China; j Department of Surgery, Division of Thoracic Surgery, Duke University Medical Center, Durham, North Carolina, USA

**Keywords:** immunotherapy, lymph node dissection, non-small cell lung cancer, PD-1 blockade, postresectional recurrence

## Abstract

**Background:**

Lymph node (LN) dissection is a common procedure for non-small cell lung cancer (NSCLC) to ascertain disease severity and treatment options. However, murine studies have indicated that excising tumor-draining LNs diminished immunotherapy effectiveness, though its applicability to clinical patients remains uncertain. Hence, the authors aim to illustrate the immunological implications of LN dissection by analyzing the impact of dissected LN (DLN) count on immunotherapy efficacy, and to propose a novel ‘immunotherapy-driven’ LN dissection strategy.

**Materials and methods:**

The authors conducted a retrospective analysis of NSCLC patients underwent anti-PD-1 immunotherapy for recurrence between 2018 and 2020, assessing outcomes based on DLN count stratification.

**Results:**

A total of 144 patients were included, of whom 59 had a DLN count less than or equal to 16 (median, IQR: 11, 7–13); 66 had a DLN count greater than 16 (median, IQR: 23, 19–29). With a median follow-up time of 14.3 months (95% CI: 11.0–17.6), the overall median progression-free survival (PFS) was 7.9 (95% CI: 4.1–11.7) months, 11.7 (95% CI: 7.9–15.6) months in the combination therapy subgroup, and 4.8 (95% CI: 3.1–6.4) months in the immunotherapy alone subgroup, respectively. In multivariable Cox analysis, DLN count less than or equal to 16 is associated with an improved PFS in all cohorts [primary cohort: HR=0.26 (95% CI: 0.07–0.89), *P*=0.03]; [validation cohort: HR=0.46 (95% CI: 0.22–0.96), *P*=0.04]; [entire cohort: HR=0.53 (95% CI: 0.32–0.89), *P*=0.02]. The prognostic benefit of DLN count less than or equal to 16 was more significant in immunotherapy alone, no adjuvant treatment, pN1, female, and squamous carcinoma subgroups. A higher level of CD8+ central memory T cell (Tcm) within LNs was associated with improved PFS (HR: 0.235, 95% CI: 0.065–0.845, *P*=0.027).

**Conclusions:**

An elevated DLN count (cutoff: 16) was associated with poorer immunotherapy efficacy in recurrent NSCLC, especially pronounced in the immunotherapy alone subgroup. CD8+Tcm proportions in LNs may also impact immunotherapy efficacy. Therefore, for patients planned for adjuvant immunotherapy, a precise rather than expanded lymphadenectomy strategy to preserve immune-depending LNs is recommended.

## Introduction

Highlights

- Immunotherapy was of efficacy in postresectional recurred non-small cell cell lung cancer.
- Dissected lymph nodes (LNs) over 16 was related to a poor response to immunotherapy.
- The beneficial effect of preserving LNs was particularly evident in immunotherapy alone subgroups.
- Preserved LNs with a high Tcm proportion was prone to better immunotherapy effectiveness.
- ʻImmunotherapy-drivenʼ LN dissection strategy is fit for patients planned for ICIs.

Postresectional recurred non-small cell lung cancer (NSCLC) is usually systemic and considered to be equivalent to stage IV disease, with a relatively poor postrecurrence survival ranging from 8 to 14 months^1,2^. In treating postresectional recurred NSCLC, the fact that targeted therapy^3^, chemo-radiotherapy^4^ could significantly improve OS and progression-free survival (PFS), has been confirmed by several randomized trials and systematic reviews. Immunotherapies that target the interaction of programmed cell death 1 (PD-1) with its ligands, PD-L1 and PD-L2^5^, have transformed the treatment strategies of advanced NSCLC^6–8^; however, it has not been properly studied whether survival benefits can be achieved by anti-PD-1 immunotherapy in the salvage therapy setting for postresectional recurred NSCLC.

There are debates on the value of dissected lymph nodes (DLN) count regarding disease staging^9,10^, metastasis risk, long-term survival^11^, and consequences for treatment options. Our team^12^ previously analyzed 38 806 cases from the SEER database and 5706 cases from the Chinese multicenter database and observed that more lymph nodes (LNs) examinations (>16) presented a better OS in stage N0 NSCLC. However, the survival benefit of more LN dissection became smaller when DLN count further increased, suggesting a harmful impact of expanded lymphadenectomy. Nevertheless, so far, no studies have examined the role of DLN count in the immunotherapy setting. On the one hand, an extensive LN dissection can help detect occult node metastasis, deliver proper adjuvant treatment, and thus improve long-term survival. On the other hand, these advantages might be offset by concerns regarding immune impairment. Expanded dissection of tumor-draining lymph nodes (TdLNs) may lead to immune impairment given the integral role these nodes play as the primary sites of tumor antigen exposure^13^ and subsequent antigen-specific immune activation^14,15^. Fransen *et al*.^16^ and Fear *et al*.^17^ found that surgical resection of TdLNs completely abrogated therapy-induced tumor regressions. van Pul *et al*.^18^ enrolled patients receiving primary melanoma excision while keeping TdLNs intact, and found that intradermal administration of anti-CTLA4 blockade led to decreased Treg and MDSC, and induced T cell activation in TdLN. Dammeijer *et al*.^14^ demonstrated that PD-1/PD-L1 interactions in TdLN but not in primary tumor correlate with prognosis. Rahim *et al.* ^19^ also reported that progenitor exhausted CD8+ T cells (Tpex) were abundant in uninvolved LNs and can mediate responses to immune checkpoint blockades (ICBs). Recently, our team has demonstrated the essential role of intact-structure TdLNs for maturation of intratumoral tertiary lymphoid structures^20^. Based on the compelling evidence presented in the aforementioned literature, we postulate a hypothesis suggesting a potential correlation between the strategy of LN dissection and the therapeutic efficacy of immunotherapy, warranting further investigation and validation in clinical settings. Patients with postresectional recurred NSCLC, who have possibly undergone systematic lymphadenectomy in the initial surgery, represent an optimal study population for investigating this research topic.

This multi-institutional retrospective study aims to demonstrate the immunological implications of LN dissection in the quantitative level for NSCLC patients with validations from multicenters, and propose a novel concept of ʻimmunotherapy-drivenʼ LN dissection strategy.

## Material and methods

### Study design and patient population

This is a multi-institutional retrospective cohort study. We retrospectively reviewed the medical records of NSCLC patients who underwent tumor resection and received anti-PD-1 therapy for postresectional recurrence from January 2018 through December 2020 from four hospitals in China. Patients eligible for enrollment met the following inclusion criteria: (1) histopathologically confirmed diagnosis of NSCLC; (2) having undergone NSCLC surgery with or without LN dissection; (3) being diagnosed as NSCLC recurrence substantiated by either imaging evidence or histopathological confirmation through repeat biopsy during the follow-up period; (4) having detailed medical records documenting the initial surgical procedure and the recurrence events; and (5) with curative-intent or tumor-controlling-intent PD-1 inhibitors for recurred disease. The exclusion criteria: (1) patients with a biopsy-proven or suspected metachronous multiple primary cancer; or (2) patients underwent second complete tumor resection for the recurrence diseases. Clinicopathologic features included sex, age, smoking history, surgical procedure, dissected LN count, driver gene mutation status, the pathologic diagnosis of TNM stage, adjuvant therapy, immunotherapy regimens for recurrence controlling, treatment cycle number, and recurrence information of the included patients were extracted from the electronic hospital information system. The optimal number of DLNs was determined by fitting the curves of hazard ratios (HRs; PFS) for each DLN count using a LOWESS smoother^21^. Structural break points were identified using the Chow test^22^. The outcomes were then compared after stratifying according to the DLN count. Subgroup analysis was performed to adjust for the effect of DLN count on immunotherapy response.

This retrospective cohort study was registered at the Research Registry (UIN: researchregistry9156). The Strengthening the Reporting of Cohort Studies in Surgery (STROCSS, Supplemental Digital Content 1, http://links.lww.com/JS9/B125) guidelines were followed to write this report^23^. This study was conducted in accordance with the Declaration of Helsinki and all of the applicable local laws and regulations. Approval for the protocol was obtained from the Institutional Review Board at the four participated hospitals (No. 0122/2020) (2020-09-11).

### Nodal dissection procedure and pathological assessments

All surgical procedures were performed using a standardized approach by certified and experienced surgeons in each of the four participating hospitals. LNs dissection involved identifying the drainage pathways and removing corresponding compartments en-bloc with adipose tissue. LNs were classified according to the International Association for the Study of Lung Cancer (IASLC) nodal map. Systematic lymphadenectomy refers to as complete as possible LN removal following established anatomical boundaries, typically including dissection of stations 2, 4, 7, 8, 9, and 10 during a right-side surgery and of stations 4, 5, 6, 7, 8, 9, and 10 during a left-side surgery. Selective lymphadenectomy and systematic sampling are the only acceptable alternatives in clinically N0 patients. Selective lymphadenectomy refers to lobe-specific nodal dissection, which means removal of interlobar (station 11) and hilar (station 10) LNs by the surgeon, as well as a minimum of three mediastinal stations according to the tumor location but including subcarinal LNs (station 7) in all cases. The surgical procedures of intraoperative lymphadenectomy were in line with the European Society of Thoracic Surgeons (ESTS) guideline^24,25^.

All resected LN specimens were sent for pathological evaluation. Pathological assessment for resected LNs was carried out by at least two expert pathologists. To ensure a standardized pathological evaluation^26^, participating pathologists received training in uniform evaluation criteria. Segmental and subsegmental LN stations 13 and 14 were not routinely retrieved, and were also not included in the calculation of the LN number. Pathologists also employed a rigorous process for screening LN fragments based on LN size and boundaries. And that calculation of the LN number only included structurally intact LNs and those composed of identifiable fragments. The evaluation outcomes from each hospital were aggregated to establish an integrated dataset, which was subsequently analyzed in our investigation.

### Oncological treatment regimens for postresectional recurrence

The selection of therapies was in line with the current NCCN guidelines^27^ and were assessed by a multidisciplinary team of thoracic surgeons and oncologists. The first-line treatment consisted of PD-1 inhibitors (pembrolizumab, nivolumab, toripalimab, tislelizumab, camrelizumab, or sintilimab) combined with platinum-based doublet chemotherapy, with or without antiangiogenesis treatment, administered every 21 days^27^ for those that had negative test results for EGFR, ALK, or ROS1 genetic variants, regardless of PD-L1 expression. Second-line immunotherapy was optional for patients with EGFR, ALK, or ROS1 mutated NSCLCs that had a poor response to TKI therapy. This immunotherapy regimen was similar to that for metastatic NSCLC recommended by the NCCN guidelines^27^, and was applied to patients based on the evidence from previous studies^28,29^. The duration of immunotherapy was continuously monitored by our clinical team, patient’s tolerance to the medication, adverse events, and response to treatment were considered in making decisions regarding treatment length.

### Endpoints and follow-up

The endpoint was PFS, which was defined as the duration from the first dose of anti-PD-1 blockade to disease progression or death. Follow-up data were gathered using electronic care records, institutional databases, and telephone call. All patients were regularly followed up until mortality or the last visit until 30 June 2021. Recurrence was diagnosed on the basis of physical examination and/or imaging findings, and the diagnosis was histologically confirmed when clinically feasible. Local recurrence was defined as tumor recurrence in the ipsilateral lung or LNs, with distant metastasis referring to recurrence in the contralateral lung or LNs, or distal organs such as the brain, liver, or bone.

### Bulk RNA-sequence of resected LNs

A total of 45 resected LN tissues from 26 NSCLC patients who underwent surgical resection in one of the participated centers were collected for RNA sequencing. Written informed consent was obtained from all patients. The FFPE material of the involved LNs (metLNs) (if any) and paired noninvolved LNs (uiLNs) were examined for LN structural integrity and FFPE quality by two experienced pathologists. To ensure sufficient RNA quantity and reduce variability, a combined extraction approach was used for LN samples with consistent metastatic status and at the same station. RNA was extracted from these FFPE using the DNA/RNA FFPE kit. The RNA library preparation workflow comprises several steps: (1) RNA enrichment, achieved through rRNA removal using a probe-based method; (2) RNA fragmentation and random primer binding; (3) first-strand cDNA synthesis using reverse transcriptase lacking RNase H activity; (4) second-strand cDNA synthesis; (5) end repair, dA tailing, and adapter ligation; and (6) PCR enrichment using a high-fidelity polymerase to amplify and select adapter-bound molecules. Of these, 7 LN samples were excluded because of low purity, and 2 were excluded due to failure of RNA library construction.

### Memory cells infiltration analysis

To quantify immune cell (especially immunological memory cells) fractions, Cibersort^30^ and xCell algorithm^31^ released on TIMER2.0 (http://timer.cistrome.org/)^32^ were utilized for analyzing bulk transcriptomic data procured from resected LNs.

### Statistical analyses

Statistical analyses were performed using SPSS 15.0 for Windows (SPSS). Differences in categorical variables were analyzed using the Fisher’s exact test. Continuous variables were compared using the *t-*test or Mann–Whitney *U* test for variables with an abnormal distribution. Survival curves were estimated using the Kaplan–Meier method, and the differences between groups were evaluated by the log-rank test. Multivariable analyses were performed to identify prognostic factors of PFS using a Cox proportional hazard model. All *P*-values are two-sided, with statistical significance set as <0.05. The forest plot was formulated using the packages of *survival and forest plot* in R version 3.0.2 (http://www.r-project.org). R packages of *funkyheatmap* was used to formulated the heatmap with baseline characteristic. Sangerbox^33^ was used to generated box plot.

## Results

### Patient characteristics

The primary cohort comprised a cohort of 46 patients at the two participated centers. A validation cohort comprised of 98 patients at the other two participated centers using the same inclusion and exclusion criteria was analyzed to further strengthen the findings from the primary cohort. The baseline characteristics of all included patients (*n*=144) are shown in the Table A.1 (Supplementary Digital Content 1, http://links.lww.com/JS9/B124). Most patients [87 (60.4%)] received combination therapy, with pemetrexed plus platinum administrated in 27/87 (31.0%) of patients, paclitaxel plus platinum in 20/87 (38.6%) of patients, and antiangiogenic therapy in 11/87 (12.6%) of patients, respectively. The median recurrence-free survival (RFS) was 23.0 months (95% CI: 19.1–26.9). All 144 patients were enrolled for the calculation of oncological and survival outcomes. This study was approved by the institutional review board of our institution, and the need to obtain written informed consent from each patient was waived.

### Effectiveness of ICB for postresectional recurred NSCLC

**Figure 1:**
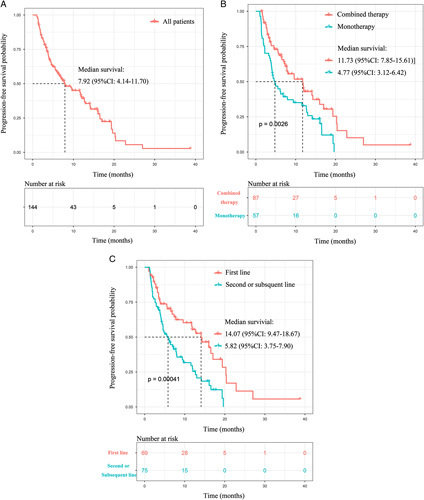
Survival outcomes of patients with postresectional recurred NSCLC treated by immunotherapy. Kaplan–Meier survival curve of progression-free Survival of (A) 144 included patients; (B) 144 included patients stratified by treatment line; (C) 144 included patients stratified by anti-PD-1 regiments.

Median PFS was 7.9 (95% CI: 4.1–11.7) months for the whole 144 patients (Fig. 1A); 11.7 (95% CI: 7.9–15.6) months in the combined therapy subgroup, 4.8 (95% CI: 3.1–6.4) months in the immunotherapy alone subgroup (Fig. 1B); 14.1 (95% CI: 9.5–18.7) months in first-line treatment subgroup, and 5.8 (95% CI: 3.8–7.9) months in subsequent-line treatment subgroup (Fig. 1C), respectively. The ORR was 36/144 (25.0%), with 82 (56.9%) patients diagnosed with stable disease (SD) and 26 patients (18.1%) with progression disease (PD). (Table A.2, Supplementary Digital Content 1, http://links.lww.com/JS9/B124).

### Impact of DLN count on ICB efficacy

**Figure 2:**
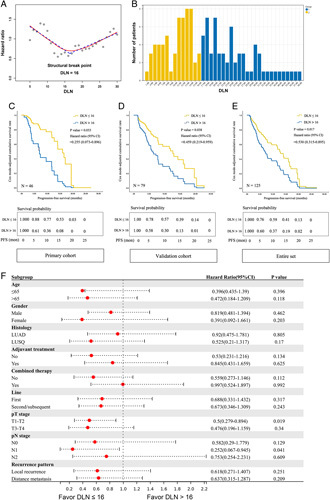
Impact of DLN count on PD-1 inhibitors efficacy. (A) Fitting curves to determinate the structural break point of DLN count for post-recurrent PD-1 blockade treatment. (B) Distribution of the number of DLN in the entire set. Multivariable Cox model adjusted cumulative progression-free survival in postoperative recurred NSCLC patients treated by PD-1 inhibitors stratified by DLN count of (C) Primary cohort; (D) Validation cohort; (E) Entire set. Adjusted variables included in the Cox model: pT stage, pN stage, age (cutoff: 65 years old), lines, regiments (anti-PD-1 combination therapy or anti-PD-1 immunotherapy alone), histology, adjuvant treatment (with or without), treatment cycles, and DLN count. (F) Forest plot of Cox-adjusted hazard ratio (95% CI) of DLN count>16 (with DLN count≤16 as reference) in different subgroups (age, histology, adjuvant treatment, regiment, line, metastasis stage).

After excluding 19 patients who lacked information on DLN count, 125 patients (primary cohort: *n*=46; validation cohort: *n*=79) were included in the analysis to assess the impact of DLN count on ICB efficacy, with the baseline characteristic shown in the Table 1. Figure 2A shows the LOWESS fitting curve and corresponding structural break points for the Cox-adjusted-HR of PFS. The HR value reached its minimum when the cutoff value was 16; therefore, the optimal cutoff value was determined as 16. The distribution of patients corresponding to different DLN count is shown in Figure 2B. The median DLN count was 18, and the majority of patients had DLN counts ranging from 4 to 27. Using a cutoff point of 16 for DLN count, there were 59 patients in the DLN less than or equal to 16 subgroup, while the DLN greater than 16 subgroup comprised 66 patients.

**Table 1:** Baseline characteristics of patients with postresectional recurred non-small cell lung cancer administrated with anti-PD-1 immunotherapy in the primary cohort, the validation cohort, and the entire set stratified by dissected lymph node (DLN) number (cutoff: 16).

	Entire set			Primary cohort			Validation cohort		
Characteristic (%)	DLN≤16 (*n*=59)	DLN>16 (*n*=66)	*P*	DLN≤16 (*n*=19)	DLN>16 (*n*=27)	*P*	DLN≤16 (*n*=40)	DLN>16 (*n*=39)	*P*
Age (median [IQR])	61 [54– 66]	62 [55– 66]	0.683	60 [53– 66]	58 [54– 66]	0.789	62 [54– 66]	63 [57– 66]	0.450
Age									
≤65	42 (71.19)	48 (72.73)	1	14 (73.68)	19 (70.37)	1	28 (70.00)	29 (74.36)	0.856
>65	17 (28.81)	18 (27.27)		5 (26.32)	8 (29.63)		12 (30.00)	10 (25.64)	
Sex									
1	46 (77.97)	54 (81.82)	0.754	17 (89.47)	22 (81.48)	0.744	29 (72.50)	32 (82.05)	0.457
2	13 (22.03)	12 (18.18)		2 (10.53)	5 (18.52)		11 (27.50)	7 (17.95)	
Smoking history									
no	30 (50.85)	33 (50.00)	1	9 (47.37)	15 (55.56)	0.804	21 (52.50)	18 (46.15)	0.735
yes	29 (49.15)	33 (50.00)		10 (52.63)	12 (44.44)		19 (47.50)	21 (53.85)	
Histology									
LUAD	40 (67.80)	37 (56.06)	0.356	9 (47.37)	15 (55.56)	0.337	31 (77.50)	22 (56.41)	0.125
LUSQ	15 (25.42)	21 (31.82)		10 (52.63)	10 (37.04)		5 (12.50)	11 (28.21)	
Other	4 (6.78)	8 (12.12)		0 (0.00)	2 (7.41)		4 (10.00)	6 (15.38)	
Surgical procedure									
Limited	3 (5.08)	1 (1.52)	0.533	2 (10.53)	1 (3.70)	0.752	1 (2.50)	0 (0.00)	1
Standard	56 (94.92)	65 (98.48)		17 (89.47)	26 (96.30)		39 (97.50)	39 (100.00)	
LN count (median [IQR])	11 [7– 13]	23 [19– 29]	<0.001	11 [6–14]	23 [19–27]	<0.001	11 [7–13]	23 [19–30]	<0.001
EGFR mutation									
Mutated	4 (6.78)	6 (9.09)	0.825	0 (0.00)	1 (3.70)	0.630	4 (10.00)	5 (12.82)	0.715
Wild type	49 (83.05)	52 (78.79)		4 (21.05)	7 (25.93)		30 (75.00)	26 (66.67)	
Non specific	6 (10.17)	8 (12.12)		15 (78.95)	19 (70.37)		6 (15.00)	8 (20.51)	
p-T stage									
T1	23 (38.98)	21 (31.82)	0.850	5 (26.32)	6 (22.22)	0.496	18 (45.00)	15 (38.46)	0.487
T2	22 (37.29)	26 (39.39)		6 (31.58)	11 (40.74)		16 (40.00)	15 (38.46)	
T3	9 (15.25)	12 (18.18)		5 (26.32)	9 (33.33)		4 (10.00)	3 (7.69)	
T4	5 (8.47)	7 (10.61)		3 (15.79)	1 (3.70)		2 (5.00)	6 (15.38)	
p-N stage									
N0	30 (50.85)	27 (40.91)	0.067	10 (52.63)	8 (29.63)	0.265	20 (50.00)	19 (48.72)	0.073
N1	7 (11.86)	19 (28.79)		3 (15.79)	8 (29.63)		4 (10.00)	11 (28.21)	
N2	22 (37.29)	20 (30.30)		6 (31.58)	11 (40.74)		16 (40.00)	9 (23.08)	
Pathological stage									
I	21 (35.59)	17 (25.76)	0.284	6 (31.58)	3 (11.11)	0.210	15 (37.50)	14 (35.90)	0.428
II	11 (18.64)	18 (27.27)		4 (21.05)	9 (33.33)		7 (17.50)	9 (23.08)	
III	27 (45.76)	29 (43.94)		9 (47.37)	15 (55.56)		18 (45.00)	14 (35.90)	
IV	0 (0.00)	2 (3.03)		0	0		0 (0.00)	2 (5.13)	
Adjuvant therapy									
No	25 (42.37)	18 (27.27)	0.113	11 (57.89)	5 (18.52)	0.014	14 (35.00)	13 (33.33)	1
Yes	34 (57.63)	48 (72.73)		8 (42.11)	22 (81.48)		26 (65.00)	26 (66.67)	
PD-1 inhibitors									
Nivolumab	7 (11.86)	11 (16.67)	0.847	4 (21.05)	4 (14.81)	0.558	3 (7.50)	7 (17.95)	0.581
Pembrolizumab	12 (20.34)	13 (19.70)		4 (21.05)	6 (22.22)		8 (20.00)	7 (17.95)	
Sintilimab	10 (16.95)	13 (19.70)		5 (26.32)	5 (18.52)		5 (12.50)	8 (20.51)	
Camrelizumab	5 (8.47)	8 (12.12)		4 (21.05)	6 (22.22)		1 (2.50)	2 (5.13)	
Tislelizumab	9 (15.25)	8 (12.12)		0 (0.00)	3 (11.11)		9 (22.50)	5 (12.82)	
Toripalimab	10 (16.95)	10 (15.15)		0 (0.00)	2 (7.41)		10 (25.00)	8 (20.51)	
Nonspecific	6 (10.17)	3 (4.55)		2 (10.53)	1 (3.70)		4 (10.00)	2 (5.13)	
Cycle number [mean (SD)]	4.48 (4.39)	5.96 (4.48)	0.065	6.11 (5.69)	6.70 (5.18)	0.713	3.70 (3.44)	5.44 (3.92)	0.040
Lines									
First	25 (42.37)	39 (59.09)	0.092	14 (73.68)	24 (88.89)	0.345	11 (27.50)	15 (38.46)	0.425
Second or later	34 (57.63)	27 (40.91)		5 (26.32)	3 (11.11)		29 (72.50)	24 (61.54)	
Combination strategy									
Combined	31 (52.54)	46 (69.70)	0.074	10 (52.63)	22 (81.48)	0.077	21 (52.50)	24 (61.54)	0.559
Monotherapy	28 (47.46)	20 (30.30)		9 (47.37)	5 (18.52)		19 (47.50)	15 (38.46)	
Metastasis stage (recurrence)									
M0	20 (33.90)	32 (48.48)	0.346	13 (68.42)	19 (70.37)	0.679	7 (17.50)	13 (33.33)	0.437
M1a	16 (27.12)	11 (16.67)		2 (10.53)	1 (3.70)		14 (35.00)	10 (25.64)	
M1b	5 (8.47)	5 (7.58)		0 (0.00)	1 (3.70)		5 (12.50)	4 (10.26)	
M1c	18 (30.51)	18 (27.27)		4 (21.05)	6 (22.22)		14 (35.00)	12 (30.77)	
Brain metastasis									
No	54 (91.53)	58 (87.88)	0.709	19 (100.00)	25 (92.59)	0.632	35 (87.50)	33 (84.62)	0.964
Yes	5 (8.47)	8 (12.12)		0 (0.00)	2 (7.41)		5 (12.50)	6 (15.38)	
Bone metastasis									
No	44 (74.58)	58 (87.88)	0.092	15 (78.95)	25 (92.59)	0.364	29 (72.50)	33 (84.62)	0.300
Yes	15 (25.42)	8 (12.12)		4 (21.05)	2 (7.41)		11 (27.50)	6 (15.38)	
Liver metastasis									
No	56 (94.92)	57 (86.36)	0.188	19 (100.00)	23 (85.19)	0.221	37 (92.50)	34 (87.18)	0.681
Yes	3 (5.08)	9 (13.64)		0 (0.00)	4 (14.81)		3 (7.50)	5 (12.82)	

Figure 2C, Figure 2D, and Figure 2E depict the multivariable Cox-adjusted cumulative PFS curves for patients in the primary cohort, validation cohort, and entire set, respectively. Table 2 presents the results of multivariable Cox-analyses assessing the HRs of various prognostic factors for PFS. The potential prognostic factors examined include age, histology, pT stage, pN stage, DLN count, adjuvant treatment, treatment line, combined therapy, and number of treatment cycles. Notably, DLN count less than 16 is associated with a shortened PFS in all cohorts [primary cohort: HR=0.26 (95% CI: 0.07–0.89), *P*=0.03]; [validation cohort: HR=0.46 (95% CI: 0.22–0.96), *P*=0.04]; [entire cohort: HR=0.53 (95% CI: 0.32–0.89), *P*=0.02] (Fig. 2C–E, Table 2). And the survival benefit of DLN count less than or equal to 16 was not influenced by the pattern of recurrence, whether it was local or distant recurrence (Fig.A.1, Supplemental Digital Content 1, http://links.lww.com/JS9/B124). A multivariable logistic analysis of ORR in 144 patients adjusted for age, sex, histology, pathological T and N stage of the primary tumor, adjuvant treatment, and cycles of PD-1 inhibitors was also performed, with results demonstrated in the Table A.3, Supplemental Digital Content 1, http://links.lww.com/JS9/B124, demonstrated an odds ratio of DLN count less than or equal to 16 for ORR was 1.78 (95% CI: 0.70–4.51).

**Table 2:** Multivariable Cox regression of predictors for progression-free survival in postoperative recurred non-small cell lung cancers treated by anti-PD-1 immunotherapy (Sample size: 125).

	Entire set (*n*=125)		Primary cohort (*n*=46)		Validation cohort (*n*=79)	
Index	HR (95% CI)	*P*	HR (95% CI)	*P*	HR (95% CI)	*P*
Age						
≤65	Reference		Reference		Reference	
>65	2.135 [1.285–3.549]	0.003	2.271 [0.826–6.244]	0.112	1.949 [1.000–3.801]	0.050
Histology (1st and 2nd)						
LUAD	Reference	0.294	Reference	0.327	Reference	0.091
LUSQ	1.101 [0.623–1.946]	0.741	1.862 [0.619–5.602]	0.269	0.850 [0.372–1.940]	0.699
Other	2.150 [0.824–5.614]	0.118	3.607 [0.348–37.39]	0.282	3.419 [1.005– 11.631]	0.049
p-T stage (1st)						
T1	Reference	0.020	Reference	0.066	Reference	0.002
T2	0.648 [0.362–1.160]	0.144	0.308 [0.094–1.005]	0.051	0.607 [0.282–1.304]	0.200
T3	1.974 [0.995–3.919]	0.052	0.640 [0.213–1.928]	0.428	5.972 [1.963–18.175]	0.002
T4	0.761 [0.298–1.945]	0.568	2.341 [0.504–10.87]	0.278	0.359 [0.099–1.300]	0.119
p-N stage (1st)						
N0	Reference	0.119	Reference	0.424	Reference	0.076
N1	0.498 [0.252–0.984]	0.045	0.764 [0.187–3.124]	0.708	0.445 [0.184–1.078]	0.073
N2	0.937 [0.524–1.676]	0.826	1.688 [0.580–4.912]	0.337	0.423 [0.172–1.040]	0.061
DLN						
>16	Reference		Reference		Reference	
≤16	0.531 [0.315–0.894]	0.017	0.255 [0.073–0.896]	0.033	0.459 [0.219–0.959]	0.038
Adjuvant						
No	Reference		Reference		Reference	
Yes	0.635 [0.356–1.131]	0.123	0.359 [0.118–1.093]	0.071	0.733 [0.317–1.699]	0.469
Line						
First	Reference		Reference		Reference	
Second or subsequent	2.037 [1.164–3.567]	0.013	3.283 [1.134–9.50]	0.028	2.364 [1.000–5.588]	0.050
Combined therapy						
No	Reference		Reference		Reference	
Yes	0.570 [0.338–0.962]	0.035	0.968 [0.308–3.050]	0.956	0.430 [0.217–0.849]	0.015
Cycles of ICIs	0.900 [0.845–0.959]	0.001	0.850 [0.766–0.943]	0.002	0.834 [0.741–0.940]	0.003

In addition, after adjusting for statistically significant variables in the above multivariable cox model, such as lines, combined therapy, age and pT stage, the survival benefit of DLN count less than or equal to 16 seemed to be more significant in the noncombined therapy [HR (95% CI): 0.56 (0.27–1.15) vs. 1.00 (0.52–1.90) for combined therapy], no adjuvant treatment [HR (95% CI): 0.53 (0.23–1.22) vs. 0.85 (0.43–1.66) for with adjuvant treatment] and pN1 [HR (95% CI): 0.25 (0.07–0.95) vs. 0.58 (0.29–1.78) for pN0 vs. 0.75 (0.25–2.23) for pN2], female [HR (95% CI): 0.39 (0.09–1.67) vs. 0.82 (0.48–1.39) for male], and LUSQ [HR (95% CI): 0.53 (0.21–1.32) vs. 0.92 (0.48–1.78) for LUAD] subgroups (Fig. 2F).

### Immunological memory cell landscape in resected LNs

**Figure 3:**
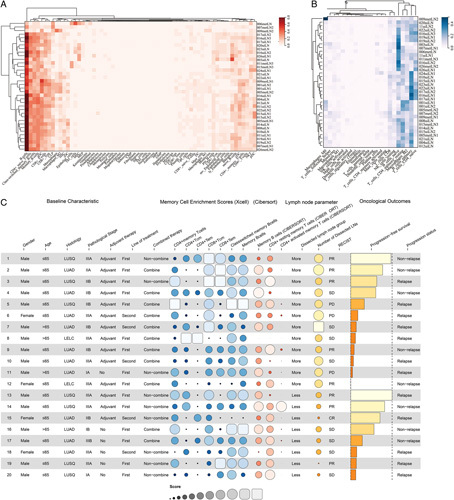
Hierarchically clustered heatmap of (A) 64×cell-calculated and (B) 22 CIBERSORT-calculated immune cell enrichment scores in resected LN specimens from 20 patients. The vertical axis represents each LN specimen while the horizontal axis represents ×Cell- or CIBERSORT-calculated immune cell enrichment scores. A deeper color on the heatmap indicates a higher enrichment score; (C) Detailed results of the 20 patients with resected LN specimens undergone bulk RNA-seq. a, Baseline characteristic, including sex, age, histology, pathological stage, adjuvant therapy, line of treatment, and whether combined therapy or not; b, ×Cell enrichment scores of CD4+ memory T cells, CD4+Tcm, CD4+Tem, CD8+Tcm, CD8+Tem, class-switched memory B cells, and memory B cells, as well as the Cibersort enrichment scores of memory B cells, CD4+ resting memory T cells, and CD4+ activated memory T cells. c, Lymph node parameters, including. DLN group (>16, ≤16), exacted DLN number. d, Oncological outcomes, including clinical response, time of progression-free survival, and progression status. LN, lymph node; RNA-seq, RNA sequencing; Tcm, central memory T cells; Tem, effector memory T cells. The size of the bubbles, represented the enrichment scores of each memory cell type, was arranged in ascending order based on the PFS.

To elucidate the underlying immunological mechanism of this phenomenon, we conducted RNA-sequence on 36 resected LN specimens obtained from 20 included patients from one of the participated hospitals. Given the extended median RFS (23.0, 95% CI: 19.1–26.9) observed in the included patients, it is reasonable to postulate that this phenomenon may be attributed to the presence of long-lived memory cells within the LNs. Hence, two hierarchically clustered heatmaps displaying immune cell enrichment scores using the xCell (Fig. 3A) and Cibersort algorithms (Fig. 3B) were generated to depict the landscape of memory cells and other immune cells within resected LNs. The detailed baseline characteristics, xCell/Cibersort enrichment scores, LN parameters, and oncological outcomes are illustrated in Figure 3C, with the quantitative data for xCell/CIBERSORT enrichment scores can be found in the Table A.5 (Supplemental Digital Content 1, http://links.lww.com/JS9/B124).

### Screening LN memory cell subsets that are associated with ICB efficacy

**Figure 4:**
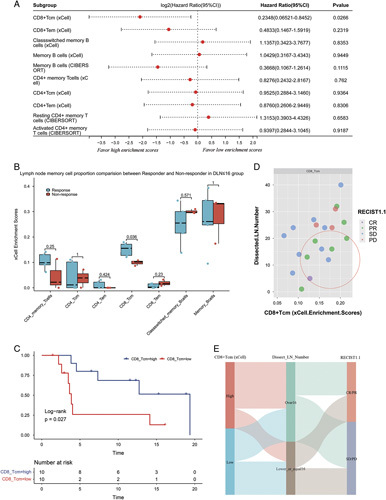
(A) Forest plot showing univariable survival analyses of LN memory cell enrichment scores. The enrichment score of each memory cell was dichotomized based on the median value. The HR (95% CI) and *P* value represent the HR and significance level of the survival advantage of the high- compared to the low-enrichment score group of each memory cell type. HR greater than 1 indicate a higher risk of progression. (B) Boxplot comparing LN memory cell enrichment scores between treatment response and nonresponse groups. The vertical axis represents the enrichment score calculated by ×Cell or CIBERSORT for various memory cell types. The horizontal axis shows the different memory cell types represented in the analysis. The blue boxes correspond to treatment response group, while the red boxes represent the nonresponse group. The *P* value for the group comparison is shown above each box. Patients who experienced a relapse within 6 months were categorized as the ‘response’ group, whereas those who did not were classified as the ‘non-response’ group. Patients with a follow-up duration of less than 6 months were excluded from the analysis. (C) Bubble plot showing the correlation between CD8+Tcm enrichment score and LN clearance, colored by RECIST1.1 response categories. Each bubble represents one patient, positioned on the plot according to their ×Cell-derived CD8+Tcm enrichment score and the number of resected LNs. The color of the bubble corresponds to the RECIST1.1 response category of the patient: purple for CR, red for PD, green for PR, and blue-gray for SD. (D) Kaplan–Meier curve plots to assess patient prognosis in CD8+Tcm high and low cohort (log-rank *P* value=0.0266). (E) The Sankey diagram visualizes the associations between LN CD8+ Tcm enrichment score, LN clearance, and RECIST1.1 response categories, using three vertical columns of connected ribbons. The leftmost column represents the ‘high LN CD8+Tcm’ and ‘low LN CD8+Tcm’ groups, divided by the median ×Cell enrichment score. The middle column displays the ‘DLN>16’ and ‘DLN≤16’ groups. The rightmost column illustrates the ‘CR/PR’ and ‘SD/PD’ groups, divided by RECIST1.1 criteria. The width of the ribbons corresponds to the patient count, with the ribbon color and column width indicating the degree of overlap between the groups. CR, complete response; HR, hazard ratio; LN, lymph node; Tcm, central memory T cells; Tem, effector memory T cells.

We dichotomized the enrichment scores of each LN memory cell into high-subgroup and low-subgroup based on the median value and the analysis revealed that high levels of LN CD8+ central memory T cells (Tcm) were with the lowest HR of 0.235 (95% CI: 0.065–0.845, log-rank *P*=0.027). Kaplan–Meier curve also demonstrated favorable prognosis in patients with high LN CD8+Tcm enrichment scores (Fig. 4C). While not statistically significant, CD8+ effector memory T cells (Tem) also exhibited an HR of 0.483 (95% CI: 0.147–1.592, *P*=0.232) (Fig. 4A). Figure 4B showed a significantly higher LN CD8+Tcm enrichment score in the response subgroup compared to the nonresponse subgroup (*P*=0.036). To simultaneously assess the impact of DLN count and LN memory cell on immunotherapy efficacy, we performed separate comparative analyses of the DLN≤16 (Fig. 4B) and DLN greater than 16 subgroups (Fig.A.2, Supplemental Digital Content 1, http://links.lww.com/JS9/B124). And a higher enrichment score of LN CD8+ Tcm, coupled with a lower count of DLNs, appeared to be associated with a higher likelihood of achieving PR or CR, as indicated by Figure 4D and Figure 4E.

## Discussions

Dissected LNs at lung cancer surgery, such as hilar and mediastinal LN, are the most common drainage sites of tumor antigens respecting NSCLC^34^. To date, clinical studies on the role of LNs for checkpoint therapy are limited. In the current study, we 1) for the first time reported the oncological and survival outcomes of patients with postresectional recurred NSCLC treated by anti-PD-1 immunotherapy, with a similar PFS compared to the first-line treatment. 2) we clarified the role of DLN count less than or equal to 16, that it can well preserve the immunological potential in LNs, thus associating with better immunotherapy efficacy. This result was adjusted by age, pT stage, DLN number, combined strategy, and cycle number, and was all qualified in the primary cohort, validation cohort, and entire set with a statistical difference; 3) elucidated that whether age, histology, adjuvant treatment, combined strategy, line and metastasis stage affect the adjusted-HR of DLN count in prediction of PFS (Fig. 4); 4) demonstrated that preserving LNs with high CD8+ Tcm proportions is related to better immunotherapy efficacy.

This multi-institutional retrospective study proved that LNs are key regulators in the therapeutic efficacy of anti-PD-1 immunotherapy for cancer treatment; and an adequate amount of DLN, not overly dissection, will be conducive to better efficacy of anti-PD-1 immunotherapy. This is in agreement with prior studies analyzing the role of TdLNs. Fransen *et al*.^16^ demonstrated that surgical resection of TdLNs completely abrogated therapy-induced tumor regressions. Similarly, Fear *et al*.^17^ also found that survival was significantly shorter in tumor-bearing mice with complete TdLN resection compared to those with intact TdLNs. Notohardjo *et al*.^18^ enrolled patients receiving primary melanoma excision and found that intradermally delivering the anti-CTLA4 blockade can decrease Treg frequencies, decrease systemic MDSC rates, activate migratory dendritic cell subsets, and induce T cell activation in the sentinel LN.

This phenomenon can be explained by several mechanisms: tumor antigens drain primarily to TdLNs via transportation by CD103+ DCs and leads to the priming of tumor-specific T-cell responses^14,15^. In addition, not only the LN structure itself, lymphatic drainage has been proven to help prime anti-tumor immunity. FTY720 is introduced as a potent immunomodulatory agent that promotes lymphocyte homing into the LNs^35^, and its use in tumor models significantly impaired the therapeutic effect of ICB^16^. Muchowicz *et al*.^36^ also verified that photodynamic therapy-induced tumor lymphatic vessel damage would weaken the ICB efficacy. Increased VEGF-C in tumor caused enhanced lymph drainage associated with a stronger immunotherapy effect^37^. Another influential factor is the presence of long-lived memory cells within the preserved LNs, which may be activated and play a role upon administration of ICBs. This study proved that high levels of LN CD8+ Tcm/Tem were associated with better ICB efficacy (HR: 0.235 and 0.483, respectively). Existing literature have indicated that protein PD-1Ab21 can promote the generation of Tscm and Tcm (CD44 high CD62L high) in the mice draining LNs^38^. Similarly, Vargas *et al.* ^39^ also reported that neoadjuvant PD-1 blockade resulted in robust proliferation of Tcm and Tscm at the TDLN along with a subsequent decrease in metastatic recurrence in 30% of mice. Under the treatment of PD-1 inhibitor, Tem and Tcm can be activated and play an anti-tumor role through transformation to the Th1 phenotype^40^, which can eventually improve anti-tumor efficacy. Evidence from mass cytometry analysis^41^ also confirmed that the abundance of Tem was significantly higher in immunotherapy responders than non-responders. These memory T cell subsets exhibit prolonged persistence and exhibit cooperative interactions with immunotherapy, tumor-specific immune cells within LNs, and suggesting a necessity of proper reservation of them.

From the surgeon’s perspective, LNs resection is a common clinical practice in many forms of cancers to determine disease staging^42,43^ and consecutive therapy options^44^. In the nonimmunotherapy setting, numerous studies have indicated a positive correlation between a high examined LN count (surpassing a certain cutoff) and improved prognosis in early-stage NSCLC^12,45,46^. As immunotherapy prevails, it is of great interest to explore the LN dissection strategy in a new scenario that patients received PD-1 blockades. The current study observed an opposite trend that higher DLN count (DLN>16, also with a similar trend of improvement in immunotherapy efficacy with 12, 13, 14, 15, 16, 17, and 20 as DLN cutoffs, see Fig. 2A) is associated with inferior immunotherapy efficacy. These findings are in contrast to the common notion of ʻmore is betterʼ idea. Hence, we proposed an adequate but not excessive dissection of LNs, that ensures complete and accurate resection of all metastases without compromising immunotherapy. While systemic LN dissection remains crucial for precise cancer staging, future considerations may involve omitting regional LN resection in appropriately chosen patients without nodal metastasis evidence or with heightened responsiveness to ICBs^47^. With adjuvant immunotherapy’s efficacy been well established in stage IB-IIIA NSCLC^48–50^, the impact of DLN count should be considered for planned recipients. To properly estimate the extent of LN dissection before surgery, it is advisable to consider LN biopsy procedures such as EBUS, incorporating evaluations of metastatic status, differentiation degree, molecular markers^51^, immune microenvironment^26,52^, and memory cell content^53,54^ to gage tumor aggressiveness. Furthermore, leveraging SUVmax values and LN size from PET/CT scans^55–57^ can aid in identifying potential metastatic LN locations and estimating LN metastatic tumor burden. These approaches offer promising avenues for furnishing surgeons with initial insights into the requisite level of DLN dissection, allowing selective sampling of LNs with minimal metastatic potential, rather than comprehensive excision, thereby preserving the LN’s immune potential prior to immunotherapy administration.

Notwithstanding the significant survival benefits of DLN greater than 16 as mentioned earlier, it is noteworthy that patients receiving immunotherapy in combination did not observe substantial benefits in the subgroup analysis [HR (95% CI): 0.56 (0.27–1.15) vs. 1.00 (0.52–1.90) for combined therapy]. This further elucidates that the benefits of preserving LNs primarily impact immunotherapy while having minimal influence on chemotherapy. This observation may also explain why, in the nonimmunotherapy era, the prevailing notion was to remove as many LNs as possible^12,45,46^, as immunotherapy was not yet considered a treatment option. Hence, the ʻimmunotherapy-drivenʼ LN dissection strategy is specifically fit for patients planned for mono-immunotherapy. For patients who have already undergone expanded LN dissection, combination immunotherapy is strongly recommended as an adjuvant consolidation treatment instead of immunotherapy alone.

And compared to those without adjuvant treatment, a relatively weaker synergistic effect of LN preservation on immunotherapy was also observed in patients received adjuvant treatment (HR: 0.53 vs. 0.85). We posit that this phenomenon might be attributed to the inherent aggressiveness of tumors (even recurred despite adjuvant chemotherapy), which is associated with diminished immunotherapeutic efficacy. Consequently, for LNs preservation as a strategy solely aimed at enhancing immunotherapy, its impact may be compromised.

The study strengths included the scarcity of cases, the multi-institutional design, strict inclusion and exclusion criteria, a rigorous and standardized LN pathological processing protocol, and the exploration of immunological mechanism by conducting RNA-seq. Besides, several limitations should be acknowledged: Firstly, the issue of LN fragmentation cannot be overlooked; although most of the fragments can be sorted out and screened by the pathologists to re-calculate the correct DLN number. Secondly, for cost reasons, we performed bulk RNA sequencing on only a subset of LNs in selected patients, and these LNs may not fully represent the immune status of all LNs. Thirdly, we did not delve into the underlying mechanisms and regulatory pathways governing the therapeutic efficacy of CD8+ Tcm/Tem in immunotherapy. Prospective studies could consider employing techniques like 2-photon intravital microscopy^58^ or 3D-cell models^59^ to offer enhanced insights underlying the response of memory cells to immunotherapy and their dynamic migration within the LNs. Fourthly, we did not collect information, such as the preoperative CT/PET and preoperative LN biopsy results. Surgeons might opt for an expanded lymphadenectomy, indicated by factors like adverse preoperative biopsy results, enlarged LNs on preoperative CT/PET, and positive nodes on EBUS. Moreover, the observed association between tumor aggressiveness and diminished immunotherapy response underscores the potential for bias introduced by these unmeasured factors^60^, which potentially confound the relationship between LN dissection extent and immunotherapy effectiveness. Fifth, tumor mutation burden (TMB) and PD-L1 expression were not routinely assessed in the participating centers, particularly among patients planned for immune-chemotherapy. The lack of these data in our dataset might limits our capacity to thoroughly investigate their impact on the observed outcomes.

Taking together, our studies encourage a precise rather than excessive LN dissection strategy to ensure clearance of all metastatic LNs and simultaneously preserve immune-depending LNs. It is essential to note that the clinical data that we collected in this real-world evidence can provide valuable insights, offer scientific evidence of purposeful significance and can significantly impact the improvement of clinical practice. Besides, while this study has identified intriguing associations between the LN dissection extent and immunotherapy efficacy, we concur that these findings are derived from correlative analyses and do not establish a causal relationship. Therefore, further validation is still warranted.

## Conclusion

In contrast to the conventional notion of ʻmore is betterʼ regarding LN dissecting, our findings revealed an unexpected association between elevated DLN count (cutoff: 16) and diminished immunotherapy efficacy in postresectional recurrent NSCLC. This finding aligns with evidence from murine models, demonstrating that surgical removal of tumor-draining LNs can abolish immunotherapy-triggered tumor regressions. The advantageous impact of LNs preservation was particularly notable among patients received immunotherapy alone for recurrence. Moreover, CD8+ Tcm proportions in LNs could also influence immunotherapy effectiveness. Therefore, for patients planned for adjuvant immunotherapy, a ʻimmunotherapy-drivenʼ LN dissection strategy refers to the nonexpanded lymphadenectomy that balances the clearance of metastatic LNs while avoiding immune impairment is recommended.

## Ethical approval

This study was conducted in accordance with the Declaration of Helsinki and all of the applicable local laws and regulations. Approval for the protocol was obtained from the Institutional Review Board at The First Affiliated Hospital of Guangzhou Medical University, Shanghai Pulmonary Hospital affiliated with Tongji University, Sun Yat-sen University Cancer Center, and the Affiliated Hospital of Guangdong Medical University (No. 0122/2020) (2020-09-11).

## Consent

The written informed consent was waived due to the retrospective setting.

## Sources of funding

This research received no specific grant from any funding agency in the public, commercial, or notfor- profit sectors.

## Author contribution

H.D.: investigation, data curation, formal analysis, writing – original draft; J.Z.: data curation, investigation; H.C.: data curation, project administration; X.C.: data curation, project administration; R.Z.: writing – reviewing and editing; F.L.: data curation; B.C.: data curation; C.L.: data curation; Q.J.: writing – reviewing and editing; C.Z.: supervision, project administration; R.H.P.: writing – reviewing and editing; G.R.: writing – reviewing and editing; A.B.: writing – reviewing and editing; C.S.H.N.: writing – reviewing and editing; T.A.D’A.: writing – reviewing and editing; C.S.: conceptualization, investigation, project administration; W.L.: conceptualization, methodology, writing – reviewing and editing; J.H.: supervision, project administration; B.Z.: conceptualization and methodology.

## Conflicts of interest disclosure

Dr René Horsleben Petersen: speaker fee from Medtronoic, AMBU, Medela, AstraZeneca. advisory board of AstraZeneca, Roche, MSD; Dr. Gaetano Rocco: financial relationship with Scanlan, Merck; Dr Brunelli reports personal fees from BARD, Astra Zeneca, Roche, Medtronic and Ethicon; Dr Calvin S.H. Ng is a consultant for Medtronic, Johnson and Johnson, and Siemens Healthineer. RTC. The remaining authors declare no conflicts of interest.

## Research registration unique identifying number (UIN)

Efficacy of immunotherapy and immunological implications of lymphadenectomy in postresectional recurred non-small cell lung cancer: a retrospective, multi-institutional, cohort study https://www.researchregistry.com/register-now#userresearchregistry/registerresearchdetails/648d49c6b5e9fa0029dfefe0/researchregistry9156.

## Guarantor

Wenhua Liang MD, and Jianxing He MD, phD, Department of Thoracic Oncology and Surgery, The First Affiliated Hospital of Guangzhou Medical University, State Key Laboratory of Respiratory Disease and National Clinical Research Center for Respiratory Disease, Guangzhou, People’s Republic of China. Tel.: +86 20 83337792; fax: +86 20 83350363. E-mail: liangwh1987@163.com/drjianxing.he@gmail.com

## Data availability statement

Deidentified participant-level dataset and statistical code will be made available for collaborative research projects, on request of the chief investigator (liangwh1987@163.com).

## Provenance and peer review

Not commissioned, externally peer-reviewed.
